# Label-Free Microcavity Biosensors: Steps towards Personalized Medicine

**DOI:** 10.3390/s121217262

**Published:** 2012-12-13

**Authors:** Dragos Amarie, James A. Glazier

**Affiliations:** Biocomplexity Institute and Department of Physics, Indiana University, Bloomington, IN 47405, USA; E-Mail: glazier@indiana.edu

**Keywords:** biosensors, microsensors, microcavity surface plasmon resonance, proteomics, interactomics, microfluidics, personalized medicine, biomarkers, single cell secretion

## Abstract

Personalized medicine has the potential to improve our ability to maintain health and treat disease, while ameliorating continuously rising healthcare costs. Translation of basic research findings to clinical applications within regulatory compliance is required for personalized medicine to become the new foundation for practice of medicine. Deploying even a few of the thousands of potential diagnostic biomarkers identified each year as part of personalized treatment workflows requires clinically efficient biosensor technologies to monitor multiple biomarkers in patients in real time. This paper discusses a critical component of a regulatory system, a microcavity optical biosensor for label-free monitoring of biomolecular interactions at physiologically-relevant concentrations. While most current biosensor research focuses on improving sensitivity, this paper emphasizes other characteristics a biosensor technology requires to be practical in a clinical setting, presenting robust microcavity biosensors which are easy to manufacture and integrate with microfluidics into flexible and redesignable platforms making the microcavity biosensors deployable for continuous monitoring of biomarkers in body fluids in the clinic, in dense 2D random arrays for high-throughput applications like drug-library screening in interactomics, and of the secretory behavior of single cells in the laboratory.

## 1. Introduction

### 1.1. Personalized Medicine

Contemporary medical practice offers few strategies to predict or prevent illness and limited stereotyped responses to clinical symptoms: diagnosis employs a narrow repertory of laboratory tests and medical history indicators, while treatments are often standardized and proscriptive rather than continuously adjusted to patient response. Doctors, scientists, and policy makers have increasingly proposed personalized medicine (PM) as a strategy to improve health maintenance, diagnostic precision, therapeutic efficacy and affordability [1,2]. PM attempts to provide a proactive strategy to replace the reactive strategy of contemporary healthcare by identifying individuals and stratifying populations susceptible to diseases before the onset of clinical symptoms, recommending prevention strategies and designing individualized treatments. However, the aims of PM have become less ambitious with time, especially since the human genome project hypothesized that genotyping could allow “personalized” therapies within a proscriptive model of treatment. Despite a number of significant successes (like BRCA1 genotyping), the genomic approach has had limited value for therapeutic optimization because most common illnesses involve the complex interplay of hundreds of genes and proteins within cells and of dozens of cell types with their tissue and organismal environment [3]. A more comprehensive PM would combine an individual’s *genomic*, *proteomic*, *metabolomic*, *interactomic* and *lifestyle* profiles to predict and prevent diseases and, when diseases do occur, to deliver therapies continuously optimized for the individual patient. Truly personalized medicine would reduce healthcare costs by preventing disease, reducing trial-and-error therapies, minimizing drug toxicity and side effects, and improving outcomes.

PM regards an individual as a *virtual map* consisting of: (1) multi-*omic* information on millions of molecular and larger-scale components (genes, proteins, metabolites, hormones, ions, small molecules, cells, tissues, organs, *etc.*), (2) their interaction networks, and (3) their dynamic responses to internal, life-style and environmental stimuli. From a PM perspective, a disease is a dynamic perturbation of such an *omics*-network [2,4] which spans a broad range of length and time scales, eventually affecting the entire organism.

### 1.2. Disease Biomarkers

Patients respond to a given disease or drugs differently, impeding accurate diagnosis and individualized titration of treatment to maximize therapeutic efficacy and minimize side effects. The complex nature of many diseases creates a need for sensitive and specific chemical reporters of patient condition (*biomarkers*) as well as inexpensive biosensor devices for diagnosing patients, monitoring treatment progress and evaluating the safety and efficiency of new therapies. Clinical practice recognized the utility of biomarkers to describe both normal and pathological conditions and began to apply them long before the term “personalized medicine” was coined (e.g., blood glucose in diabetes, troponin in heart attacks, human epidermal growth factor receptor 2 (*HER2*) in breast cancer, *etc.*) [5–7]. However, we are far from making optimal use of biomarkers. The number of biomarkers we follow is still very limited, although no single biomarker is specific enough to describe comprehensively both disease progression and patient response to treatment; e.g., some biomarkers are relevant to diagnosis, while other provide insight into disease progression. Because biomarker characteristics (e.g., concentrations of secreted biomolecules into body fluids, reaction kinetics constants, enzymatic activity, single-cells secretion profiles, *etc*.) vary from individual to individual and because disease states involve dynamic changes in levels, dynamic measurements of levels provide more revealing information than single-point measurements about wellness or disease states. Monitoring changes in biomarker characteristics will allow us to prevent disease through early diagnosis, identify patients’ susceptibility to different therapies, interactively optimize treatment for effectiveness and prevent disease relapse, while reducing time and costs related to clinical validation of therapies. Such dynamic measurements are currently available for a very limited number of targets (EKG, blood oxygen, glucose, blood pressure, temperature, and pH for patients with a central line) while point-of-care panel techniques like micro-ELISA provide single-time-point measurements only. Thus one key requirement for PM is a new biosensor technology to enable dynamic biomarker panel measurement.

A few examples may help clarify the complex issues surrounding clinical biomarker applications. In Alzheimer’s Disease (AD) the correlation of three biomarker levels (beta-amyloid 42, *Aβ42*, 4.4 kDa; total tau protein, *t-tau*, 40–60 kDa; and tau phosphorylated at position threonine 181, *p-tau*, 46–68 kDa) identifies the very early onset of the disease [8]. Monitoring the levels of these three biomarkers in the cerebrospinal-fluid (*CSF*) is complex: P-tau levels are lower and fluctuate over narrower ranges (0.8–1.5 pM in healthy individuals and 1.0–2.4 pM in AD patients) than t-tau levels (3.9–11.2 pM in healthy individuals and 10.1–25.7 pM in AD patients) and Aβ42 levels (140–180 pM in healthy individuals and 60–85 pM in AD patients). P-tau and t-tau levels are lower in healthy individuals than in AD patients, while Aβ42 levels are lower in AD patients than in healthy individuals. Aβ42 levels in healthy individuals and AD patients do not overlap, while p-tau level ranges overlap over 30% of their range [9]. Characterizing the progression of AD while accounting for observed patient-to-patient variation thus requires a panel of at least 8 biomarkers [10]. Each disease requires its own panel of biomarkers for diagnosis or monitoring: myocardial infarction diagnosis and monitoring currently employ 14 biomarkers [11], sepsis diagnosis typically employs five different biomarkers (and an additional 170 have been proposed) [12], and typical cerebral damage diagnosis assays employ six biomarkers [13]. Clearly, a single mega-panel providing a comprehensive suite of biomarkers in a single test would be optimal, especially in an emergency-room setting, where the underlying disorder may not yet have been identified.

The concentrations of biomarkers in body fluids vary significantly from disease to disease for the same individual and from individual to individual for the same disease, though typical physiologically relevant concentrations range from 0.1 pM to 10 mM; e.g., p-tau has a typical cerebrospinal fluid concentration below 1 pM; neuron-specific enolase (*NSE*, 78 kDa), a cerebral damage biomarker in serum has a concentration range from 0.1 to 4.7 nM, and is negligible in healthy individuals [13–15]; in severe septic shock, interleukin 6 (*IL-6*, 27 kDa) levels increase about 1,000 times above normal values, peaking at 18 nM [16–18]; while blood glucose deviation from a normal level of ≈4 mM indicates diabetes [19]. Thus to support a broad range of biomarker panel assays, a biosensor technology must be able to handle both a variety of body-fluid sources and a great range of biomarker concentrations.

### 1.3. Biomarkers at the Single-Cell Level

A key challenge to developing personalized treatments comes from the extraordinary cellular heterogeneity in any individual; so that any disease involves a multitude of dysfunctional molecular, genetic and environmental perturbations. The effort to unveil the pathologic mechanisms of diseases, identify novel disease biomarkers, validate biomarker diagnostic assays and their clinical utility require comprehensive, multi-scale studies from single-cell to organism.

Even under controlled, *in vitro* conditions, individual cells of the same type in the same environment are highly heterogeneous in their molecular responses. While the genome is nearly constant across an organism, the proteome and interactome vary from cell to cell and time to time. The aggregation of signals from multiple cells, which current assays require, washes out this heterogeneity and masks the differing responses of individual cells to disease states and treatments, impeding development of diagnostics and therapies. Most biomarkers in body fluids (e.g., blood, urine, sweat, cerebrospinal fluid, saliva, *etc.*) aggregate the secretory activity of thousands or millions of individual cells. Cell secretions control a wide range of functions (e.g., regulatory proteins, digestive enzymes, growth factors, cytokines, antibodies, mucus, structural proteins, extracellular matrix, *etc.*). An individual cell’s secretion is highly heterogeneous and depends on its cell-cycle phase, its interaction with other cells and its microenvironment, the disease state and the cell history [20]. While most clinical biomarker assays will likely continue to focus on aggregated samples, the ability to monitor secretory states at the single-cell level would greatly improve our understanding of the causal mechanisms of disease origin and progression, as well as allowing more effective validation of new biomarkers.

While we currently lack techniques to monitor the secretory states of individual cells, miniaturized biosensors, placed next to cells could analyze secretion by single cells. Sample physiological secretion rates range from 10–13 fg/cell/h for IL-6 in T and spleen cells [21], to 10–50 fg/cell/h for Aβ40-42 in neurons [22], to 2–40 fg/cell/h for vascular endothelial growth factor (*VEGF*, 45 kDa) in fibroblasts [23] and 1.8–12 ng/islet/h for insulin (5.8 kDa) in pancreatic tissue [24]. Such amounts are too small to measure if diluted over many micro-liter sample volumes, but would be measurable if the biosensor could be housed adjacent to the cell. In a 10 pL chamber, e.g., 10 fg/cell/h of IL-6 (222,000 molecules/h) would produce a 37 nM/h concentration [23]. Thus a very small biosensor could enable the development of clinically relevant single-cell or micro-tissue personalized diagnostic assays.

### 1.4. Clinical Utility and Biosensor Design

Personalized medicine clearly requires biosensor technologies which are practical to use in a clinical setting to monitor biomarkers. Surprisingly, while thousands of research papers every year present new biomarkers, much less effort has addressed the development of biosensor technologies oriented towards biomarker monitoring. In addition, most current biosensor research focuses on improving sensitivity [25]. However, sensitivity is often not the key factor determining whether a biosensor technology is suitable to clinical applications.

A biosensor sensitive enough to measure solute concentrations in real time is a necessary component of such a technology. However, for a dynamic measurement technology to be clinically useful, it must satisfy a number of additional criteria: it must be integrated into an instrument with a workflow that a clinician can use easily, it must be reliable and reproducible, and it must apply to a broad range of patient conditions. While many types of biosensor are highly sensitive [25], biosensors vary in their fit to other clinical needs. An ideal biosensor [26] would require very small volumes of sample to make it minimally-invasive. Since biomarkers in the blood stream span a concentration range, from 0.1 pM to 10 mM, the biosensor should allow the accurate quantification of concentrations over an extended dynamic range. Because disease characterization requires the dynamic monitoring of large panels of biomarkers, the biosensor should be small enough to allow simultaneous measurements of hundreds or thousands of biomarkers in real time. The biosensor must be robust, simple to install and have a long shelf-life, so it is convenient to use. It also needs to be easy to make and mount into an inexpensive instrument so that it is affordable in a clinical setting.

In our research, we have developed a microcavity biosensor technology which we can apply both in the laboratory, to discover biomarkers or to develop single-cell diagnostic assays, and in the clinic, to provide accurate real-time measurements of biomarkers in patients.

### 1.5. Overview of Selected Biosensor Technologies

Since a recent review has covered many technological advances in biosensors for biomarker discovery [27], we will limit our discussion to the most widely used technologies for molecular quantification of biomarkers in body fluids and at the single-cell level. Design and manufacturing complexity, and high operating and maintenance costs have prevented the application of most of these technologies in PM and point-of-care diagnostics. Size and sample volume issues have similarly impeded their use to monitor protein secretion at the single-cell level [28,29].

*ELISA* (including the *micro-ELISA* and *digital-ELISA*) is the leading technology in diagnostics and biomarker development. It is both sensitive and specific even in complex body fluids (with a detection limit of 0.03 pg/mL for IL-6 or 50 aM for prostate-specific antigen for digital-ELISA). However, its batch mode sandwich immunoassay takes 5-6 h, so it cannot monitor reaction kinetics in real time [30,31]. It also requires fairly large sample volumes of > 50 μL, ruling out single-cell analysis. Cost is high, at ≈$500/experiment (less for micro-ELISA). Throughput is limited and redesign difficult.

*Mass spectrometry* (MS) and *high-performance liquid chromatography* (HPLC) are primarily tools for biomarker discovery, though microfluidic MS and LC are beginning to make their way into clinical applications. Classic technologies require sample volumes of 5–10 μL, while newer technologies like capillary electrophoresis-electrospray ionization-mass spectrometry requires only 6.7 nL [32]. MS cannot analyze molecules larger than 2 kDa, is expensive in its bench-top form (≈$250,000) and relatively insensitive, with a detection limit of 4 fg (for evaporated samples) [33]. HPLC can detect any size molecule with a detection limit of 10 ng/mL [34]. Neither allows easy redesign, sample recovery, high-throughput or single-cell analysis.

*Microcantilevers* integrate well with microfluidics and can detect sample masses of the order of 1 pg. Their relatively large size, (20–100) μm wide × (100–500) μm long [35] limits measurement speed, and number of sensors per chip and makes them sensitive to artifacts due to nonspecific binding in complex body fluids [36]. Single-cell secretion analysis is possible in principle, but cantilevers lose sensitivity when miniaturized.

*Electrochemical* biosensors are diverse in size and operation, using multiple physical principles for detection (e.g., amperometric, calorimetric, potentiometric and conductometric) with typical detection limits of 450 aM for DNA molecules and 4.0 fg/mL for proteins [29]. They have been widely used for single-cell secretion monitoring, but usually require very complex ligands and conductive polymer coatings instead of the simple antibodies of other label-free techniques, increasing costs and limiting the range of molecules that they can detect [28].

*Whispering-Gallery-Mode (WGM)* optical biosensors are spherical, cylindrical or toroidal structures 50 μm to 1 mm in size [37–42], that can dynamically monitor molecular interactions. They require complex optical instruments to house them, impeding their use in portable medical devices, and their relatively large size prevents their use for single-cell detection of multiple secreted molecules. As with other optical detection techniques, secondary antibody amplification improves selectivity and sensitivity, allowing a detection limit of 6.5 pM, at the cost of reduced speed, loss of dynamic response and a narrower range of targets per device [43].

*Backscattering Interference* (BSI) uses a ≈30 μm channel to detect solutes at concentrations of ≈8 pg/mm^2^, either with the target molecule fixed to the channel’s surface or with both species free in solution [44,45]. It integrates well with microfluidics, but scales poorly because detecting the optical interference pattern which the sensor emits requires relatively large linear sensor arrays. BSI could, in principle, allow single-cell secretion monitoring.

*Surface Plasmon Resonance* (SPR) [46–50] is the leading technique in life-science research for label-free kinetic interaction analysis for biomolecules larger than 200 Da. It provides a detection limit of 1 pg/mm^2^[51]. Classical SPR biosensors use a large (≈1 mm^2^), thin (≈50 nm) flat gold film to support a surface-bound plasmon wave whose properties depend on the refractive index of the medium in contact with the sensor’s gold surface. SPR biosensors respond almost instantly when biomolecules bind to or accumulate on the sensor’s surface, allowing real-time monitoring of molecular interactions and computation of the kinetic reaction constants important in interactomics. Coating the sensor with dextran improves sensitivity at the cost of slower response time. High throughput versions of classical SPR integrate hundreds of smaller biosensors (>∅250 μm) onto a single chip, but smaller sensors are less sensitive to small molecules, setting a lower detection limit of >5 kDa. SPR’s supporting optics and fluidics are complex, limiting them to expensive desktop instruments (≈$250,000), though lower sensitivity instruments are about five-fold less expensive. The cost per experiment is also fairly high ≈$300/experiment. SPR requires large sample volumes, preventing single-cell-level analysis.

Many other *optical biosensor* technologies [52,53] are under development, but not yet widely deployed in biomedical applications. Their mechanisms of detection include molecular spectroscopy using IR absorption [54], UV absorption [55] and Raman scattering [56,57]; measurement of fluorescence intensity [58], quenching [59] and resonance energy transfer [60]; guided-mode spectroscopy using optical fibers [61]; interferometry using resonant mirrors [62] and grating couplers [63,64]; colorimetry [65]; reflectometry [66]; and ellipsometry [67].

An additional group of biosensing assays used in basic research and for histology and pathology analyses identify intra or extracellular protein levels *in situ* in cells or tissues. Such technologies are not easily configured to work in portable medical devices.

*Limiting Dilution Analysis (LDA)* determines the frequency of protein-secreting cells in a population with sensitivity comparable to ELISA. Secreted protein can sometimes be detected shortly after stimulation, but the assay commonly takes days and the assay destroys the cells [68–75].

*Enzyme-linked immunosorbent spot* (ELISPOT) and *in situ Cell Secretion Assays* (CSA) [76] also assess cells’ protein production [77–84]. They are 10–200 times more sensitive than ELISA [85,86] and fast, but can only measure one protein per assay. Both require fluorescent labels and destroy the analyzed cells, preventing dynamic measurements and cell recovery.

*Flow cytometry* (FC) characterizes a few proteins at high speed in large populations of cells using specific fluorochromes. It allows cell recovery, but is only semi-quantitative, cannot monitor biomolecular interactions directly and does not work with intact microtissues. FC does allow indirect monitoring of cell secretion by treating with brefeldin A or monensin, which irreversibly collapses the Golgi complex, causing normally secreted proteins to accumulate inside cells and allowing their detection [87–97], at the cost of killing the cells.

*Fluorescent-tagged fusion proteins* require genetic modification of the cells to be analyzed, which is not practical in a human patient and limits the number of detectable species. Monitoring is dynamic, but the production, secretion and diffusion rates of the fusion protein differ from those of the native protein. Fluorescent labeling works well with FC to allow cell sorting and recovery.

*Immunohistochemical staining* (IHS) determines the localization and concentrations of at most a few molecular species. Since vital stains are not available for most molecules, IHS usually employs fixed cells or tissue sections, preventing dynamic measurements and cell recovery. Diffusible molecules are often lost during processing and undetectable to IHS. Sensitivity is similar to ELISA.

*In situ hybridization* (ISH) provide semi-quantitative visualization of mRNA levels in cells in fixed tissues [98–102], while *polymerase chain reaction* (PCR) detects DNA and RNA levels in lysates of single cells. Unfortunately, mRNA concentration correlates only weakly with the intracellular concentrations of the corresponding molecules and has almost no correlation with extracellular concentrations, so it is inappropriate for secretion studies [103,104].

### 1.6. Microcavity Biosensors

**Figure 1.**
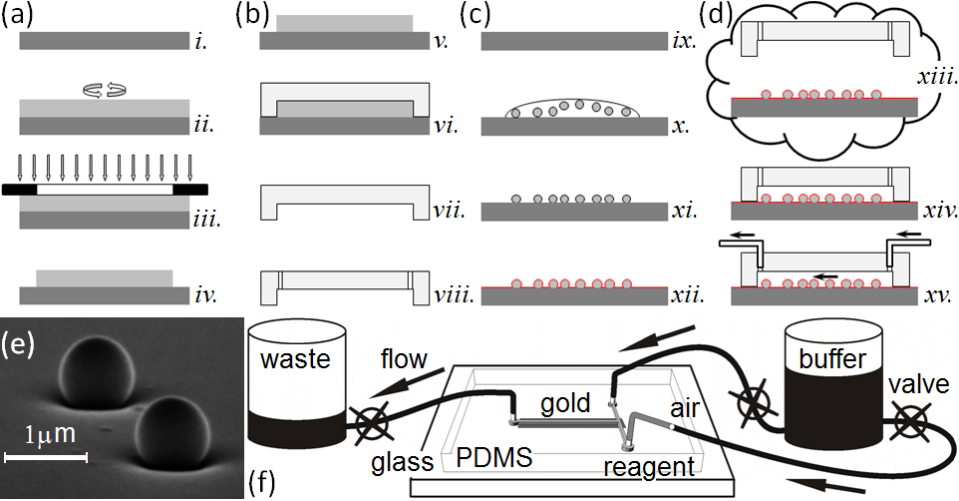
Main steps in microsensor chip fabrication. (**a**) Microfluidic master mold preparation: *i.* cleaning the glass substrate for the microfluidic mold blank; *ii.* spin-coating the blank with photoresist and prebaking; *iii.* exposing the photoresist through a mask to UV light and postbaking; *iv.* removing any unexposed photoresist from the blank with SU-8 developer and drying. (**b**) Microfluidic housing preparation: *v.* cleaning the microfluidic mold under *N*_2_; *vi.* casting the microchannels in the housing in PDMS; *vii.* removing the PDMS housing from the mold; *viii.* cutting access holes in the microfluidic housing to allow connections to external fluid sources. (**c**) Microsensor substrate preparation: *ix.* cleaning the cover-glass; *x.* dispensing the nanosphere solution onto the coverglass; *xi. d*rying the coverglass under low vacuum to attach the nanospheres to the coverglass; *xii.* sputtering gold over the nanospheres and glass surface to make a microsensor substrate. (**d**) Microsensor chip preparation: *xiii.* plasma cleaning the PDMS housing and microsensor substrate; *xiv.* joining the coverglass microsensor substrate and PDMS housing to make a microsensor chip; *xv.* connecting the fluid access lines to the microsensor chip. (**e**) Scanning electron micrograph (75° tilt) showing two microsensors ≈5 μm apart on a microsensor substrate. Each microsensor consists of a 780-nm polystyrene nanosphere attached to a flat glass surface, then uniformly sputtered with a 170-nm-thick Au film over Cr. (**f**) Schematic of a microsensor chip assembly with fluid supply attached via inlet tubes. The microfluidic channels cover a gold stripe with ≈5,000 randomly distributed microsensors. Two 4-mm-long input channels bring buffer and reagent to the microsensors. We have backfilled one inlet tube so that an air bubble separates its reagent from the remaining buffer. We control flow hydrostatically using the valves indicated. Arrows show flow direction. Not to scale.

This paper presents a sensitive, label-free, dynamic optical microsensor designed to meet our biomedical utility criteria. The microsensor’s emission spectrum has characteristic spectral peaks (*microcavity resonances*) generated by *surface plasmon* (SP) waves confined to a gold cavity *wrapped around a sub-micron dielectric sphere*[105,106]. These microsensors have a number of advantages compared to the technologies we have just discussed. (1) They are both label-free, which allows dynamic measurements of concentration, and more sensitive than other label-free biosensor techniques [106]. A single microsensor can distinguish the interaction of glucose oxidase (*GOx*, 160 kDa) with its natural substrate β-d-glucose (*βDGlu*, 180 Da) from its interactions with the non-specific substrates l-glucose (*LGlu*), d-mannose and 2-deoxy-d-glucose; while a Biacore 3000 using classic SPR technology cannot. As few as 35,000 glucose-oxidase molecules (9.6 fg or 60 zmol) saturate a microsensor (about 10^6^ times fewer than the Biacore 3000 biosensor requires) allowing the microsensors to quantify secretions from individual cells. (2) Each microsensor is very small, ∅1 μm (≈2.5 μm^2^ surface area), allowing their integration into large parallel arrays inside microfluidic networks (Figure 1). Even small sample flow rates of ≈2 nL/s and sample volumes of less than 5 μL allow dynamic concentration measurements [27]. (3) The microsensors provide quantitative measurements over physiologically relevant ligand concentrations. This paper presents experiments covering a 1 nM to 100 nM range, and we have published elsewhere, experiments covering a 1 μM to 100 mM range [27]. These ranges did not approach the intrinsic sensitivity limits of the microsensors, and we are currently conducting experiments to quantify their intrinsic detection limits. (4) The microsensors are robust, simple to fabricate and easy to redesign (Figure 1(a–d)). (5) They operate in a simple optical transmission geometry, sandwiched between an optical excitation source and detector (see Section 2.6) enabling their incorporation into simple, inexpensive instruments which would be affordable in a clinical setting.

## 2. Experimental Methods

In this section, we describe the typical fabrication protocols to manufacture microsensor arrays and their assembly into simple microsensor chips for quantitative characterization. The assembled chip consists of an array of polystyrene nanospheres on glass, both coated with sputtered gold, mounted into a poly-dimethylsiloxane (*PDMS*) elastomer microchannel housing cast using a master mold. After chip fabrication, we functionalize the microsensors with a particular target protein, and then mount the chip into an optical setup for sensitivity, selectivity and durability analysis. Below, we describe our protocols for making the microfluidic masters, the microfluidic housings and the microsensor chips (made by bonding the microfluidic housing to the microsensor array). We also describe the functionalization of the microsensors and our apparatus for measuring microsensor properties. We have previously described an earlier version of these protocols in [106].

### 2.1. Microfluidic Mold Fabrication

To manufacture the master mold for the microfluidic housings, we used a SU-8 2010 photoresist (MicroChem Corp.) to cast microchannels in PDMS (Figure 1(a)). We designed a photomask for the microfluidic pattern, a T-intersection of 50 μm wide channels, using AutoCAD LT 2004 (AutoDesk, Inc.) and printed it on a high neutral density transparency (*mask*) using a high-resolution laser photoplotter (40,640 dpi, Photoplot Store).

To make the substrate blank for the mold, we cleaned glass slides (75 × 50 × 1 mm, Corning) in NH_4_OH/H_2_O_2_/H_2_O (1:1:1) at ≈75 °C for one hour or until the boiling stopped, then rinsed abundantly with DI-water, methanol and dried under a strong stream of nitrogen (80 psi, Industrial Grade, Airgas, Inc.). The mold had two SU-8 layers. The first layer (≈20 μm thick) promoted the adhesion of the channel structure to the glass. The second layer became the channel structure. We processed both layers identically, except that we exposed the first layer without a photomask. The SU-8 master height was 21.4 ± 0.3 μm (*n* = 15) measured across the structure with a stylus profiler (Dektak 6M, Veeco Instruments, Inc.).

To apply photoresist to the mold blank, we spin-coated the SU-8 (WS-400B-6NPP, Laurell Technologies, Co.) onto the glass slides by ramping at 106 rpm/s to 1,000 rpm, then holding for 30 s. We prebaked the photoresist on a digital hot-plate (732P, PMC Industries) at 65 °C for 1 min, ramping to 95 °C at 100 °C/h, and then holding for 3 min.

We then contact-printed the microfluidic pattern onto the photoresist-coated blank using UV-light (365 nm, 150 mJ/cm^2^; 205S, Optical Associates, Inc.) from a high-pressure Hg-arc lamp with an additional 360 nm band filter (fwhm 45 nm, Edmund Optics, Inc.). We postbaked the exposed photoresist at 65 °C for 1 min, ramping at 300 °C/h to 95 °C, and then holding for 1 min. We removed the unexposed photoresist by soaking for 10 min in SU-8 Developer (MicroChem Corp.), rinsed with 2-propanol (Sigma-Aldrich) and dried gently with nitrogen.

### 2.2. Microfluidic Housing Fabrication

The fabrication of individual microfluidic housings used an SU-8 master mold (Figure 1(a)) whose quality (specifically, the shape accuracy of the negatives of the channels) was crucial to the quality of the housings and indirectly depended on the quality of the photomask, the uniform deposition of the photosensitive SU-8 layers, the accuracy of the photomask registration on the SU-8 blank, and on the photoresist and SU-8 exposure and etching protocols.

We cast the housings with the microchannels in PDMS (Figure 1(b)) [107]. We mixed the PDMS polymer base and the curing agent (Sylgard 184 Silicone, Dow Corning Corp.) at a ratio of 10:1 (mass/mass) for ≈2 min. A Scotch™ Tape barrier placed around the mold held the PDMS elastomer mixture in place as we poured the mixture onto the master mold. We then placed the mold under low vacuum (≈1 Torr) for one hour to enhance channel replication accuracy, cured it at 100 °C for 30 min and immediately separated the hot polymerized PDMS housings from the master mold. This technique avoided the need for SU-8 master mold silanization [108,109].

The channel design was a T-type intersection with two 50-μm-wide inputs, one for buffer and one for reagent injection, both connecting to a *main chamber* (≈10 nL). We collected the waste at the opposite end of the main chamber. Microfluidic channels terminated in ∅2 mm disks into which we punched fluid access holes through the elastomer using 16-gauge titanium-nitride-coated needles for clean cuts. We blew dry nitrogen over the PDMS housings to remove surface debris from the punching, post-baked for 24 hours at 120 °C, to remove any volatiles or unpolymerized residues, and stored the finished PDMS housings in clean, covered Petri dishes.

### 2.3. Microsensor Array Fabrication

We built the microsensor arrays by depositing polystyrene nanospheres onto cover-glass substrates then coating them with a thin sputtered layer of Au either directly on the nanospheres and glass or over a very thin Cr attachment layer. The key properties of the microsensor array were the uniformity of the cores (nanospheres), their spatial distribution on the glass substrate and the strength of their binding to the glass substrate. The frequency and quality factor of the microcavity resonances depended on the uniformity and texture of the Au or Au/Cr coating and the detailed geometry of the metal coating at the neck where the nanospheres contact the glass, these geometrical factors in turn depended both on the deposition method and glass surface properties. To produce a narrow neck and a uniform coating thickness on the nanospheres, we used omnidirectional sputtering rather than more common directional evaporation methods to deposit the metal layers.

We fabricated the microsensors on optically transparent cover-glass (No. 1½, 50 × 24 × 0.18 mm, VWR), which we rinsed with 70% ethanol (reagent grade, Sigma-Aldrich) in nanopure water (18.2 MΩ·cm, Nanopure Life Science UV/UF, Barnstead), cleaned in NH_4_OH/H_2_O_2_/H_2_O (1:1:1) at ≈75 °C for one hour or until boiling stopped, then rinsed abundantly with nanopure water and dried under a strong stream of nitrogen (80 psi, Industrial Grade, Airgas, Inc.). We stored the substrates vertically in custom made boxes, so the substrates touched neither each other nor the walls of the box.

To fabricate a microsensor substrate, we dispensed 70 μL of a solution of polystyrene (*PS*) nanospheres (∅780 ± 5.9 nm) onto a cleaned cover-glass. We prepared the nanosphere solution by diluting a stock solution (Nanobead NIST Traceable Particle Size Standard 750 nm, Polysciences, Inc.) in methanol (ACS reagent, Sigma-Aldrich) to an estimated concentration of 10^7^ nanospheres/mL. The methanol formulation had a very low (10^−3^%) dried residue content, to avoid interfering with nanosphere adhesion to the glass substrate. We dried the cover-glass with the attached nanospheres at low vacuum (≈1 Torr) until the methanol drop evaporated, then stored each substrate, face up, in a covered Petri dish. This attachment method produced a random spatial pattern of nanospheres 1–50 μm apart strongly attached to the substrate (Figure 1(e)).

We sputtered a thin, uniform Au or Cr/Au layer on the cover-glass and attached nanospheres (K-675D Dual-Target Turbo Sputter Coater, Quorum Technologies Ltd.) through a mask placed directly in contact with the substrate, with the slit of the mask directly above the cover-glass region containing the nanospheres, to produce one rectangular metallic domain (2 × 10 mm^2^) on the substrate. We used two types of metal coatings: (1) a single Au layer (120–250 nm) sputtered under a 7.5 mTorr argon atmosphere and (2) a very thin Cr layer (2–5 nm) followed immediately by an Au layer (120–250 nm) both sputtered under a 7.5 mTorr argon atmosphere without breaking the vacuum. The layers uniformly coated the nanospheres and the flat cover-glass between them (Figure 1(c)). The Cr layer significantly improved the adhesion of the Au layer to the glass, increasing the lifetime of the microsensor assemblies without noticeably affecting the optical properties of the microsensors.

**Figure 2.**
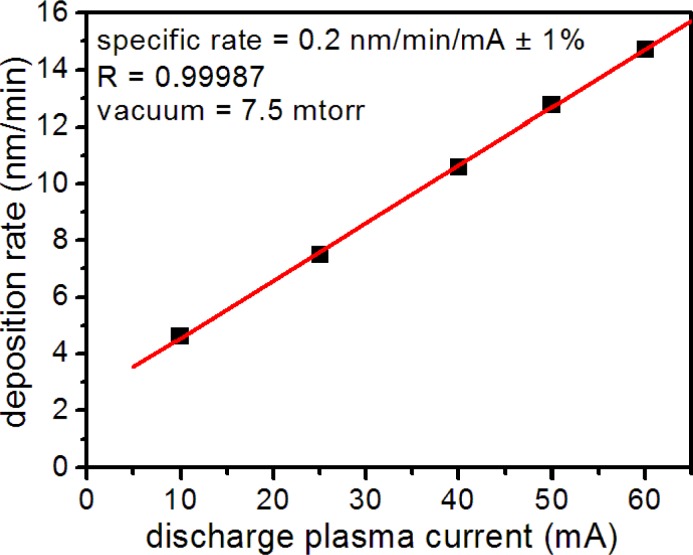
Sputtered gold deposition rate *vs.* plasma discharge currents holding the vacuum pressure at 7.5 mTorr during metal deposition. The deposition rate is linear in the discharge current with a correlation coefficient *R* = 0.999.

Metal layer quality depended on numerous factors in the sputter coater, including plasma discharge current, vacuum, deposition time and target wear. To ensure reproducible layer thickness and texture, all of which affected microsensor performance, we monitored the metal deposition rate and thickness using a quartz microbalance thickness monitor. Under constant vacuum, we found that the Au deposition rate depended linearly on the plasma discharge current (Figure 2) allowing reproducible metal deposition. For substrate production, we sputtered all nanospheres at a discharge current of 50 mA and vacuum pressure of 7.5 mTorr. Before use, we checked all microsensor substrates for consistent metal deposition by measuring the uniformity of optical transmission at numerous locations in the metal layer.

### 2.4. Microsensor Chip Assembly

To make a microsensor chip, we bonded the PDMS housing containing the microfluidic channels to the substrate supporting the microsensor arrays. While the registration tolerance for the assembly was not demanding, the bond needed be leak-free for the chips to be usable. To prepare clean, self-adhesive surfaces for bonding, we exposed the microsensor substrate to air plasma (PDC-32G, Harrick Plasma) for 3 min to remove any surface impurities, and then simultaneously exposed both the microsensor substrate and the PDMS housing to air plasma for 40 s and immediately joined them, forming a permanent, leak-free seal. This assembly, with the microsensors at the bottom of a microchannel constituted a microsensor *chip* (Figure 1(d)). The chips had two fluid input channels; a *buffer* and a *reagent* channel both feeding into a common microsensor *chamber* (0.05 × 10 mm^2^) containing more than 100 randomly distributed microsensors. Priming the chip with *running buffer* through the *waste reservoir* minimized bubble formation and uniformly wet the channels.

### 2.5. Microfluidic Flow Control

We used pressure-driven flow to bring controlled volumes and concentrations of buffer and reagent solution into contact with the microsensors in the chips (Figure 1(e)). We connected the input-channels to a common 25 mL graduated cylinder using 1.6 mm i.d. polypropylene tubing [108,109]. In-line stainless steel valves (Swagelok Company) allowed us to turn the flow on and off manually. The output line from the chamber ran to a 25 mL graduated waste-cylinder controlled by a manual valve (Figure 1(e)). The diameter of the 25 mL graduated cylinders was large enough that the fluid level did not change noticeably during the experiments, allowing us to maintain a constant flow rate. We ran buffer continuously through the tubes and chip between experiments to keep the chip free from sediment and bubbles. Before each experiment we manually backfilled the end of the reagent tube with ≈4 μL of reagent solution using a Hamilton microsyringe. To reduce flow fluctuations in the chip due to accidental vibration of the tubes, we injected an air-bubble into the inlet tube. Besides reducing mechanical coupling to the supply cylinders, the air bubble prevented reagent mixing with the buffer solution.

We maintained a constant flow rate through the chip at all times by adjusting the relative height (Δ*H*) of the liquid level in the input graduated cylinder with respect to that in the waste cylinder. A relative height of Δ*H* = 79 mm produced a flow rate of ≈0.2 nL/s through the chip, which we calculated by monitoring the movement of the air bubble inside the input tube over 20 hours.

When we mounted a new microsensor chip for use, we first thoroughly cleaned the tubes connecting to the microfluidics before connecting them to the freshly primed microsensor chip. We cleaned the tubes with DI-water, eluted with 0.1 M HCl, rinsed with DI-water, then with methanol, then DI-water, then 0.1 M NaOH, and finally with two more rounds of DI-water.

### 2.6. Optical Setup

The microsensors work in an optical transmission mode; to interrogate the microcavity resonances’ change in amplitude and frequency induced by molecular binding we mounted the microsensor chips on a custom-modified microscope which allowed separate control of the illumination and analysis of the light from individual sensors using a spectrometer/monochromator. Key factors to maintain adequate signal-to-noise during a run included controlling the illuminator intensity and maintaining the microsensor centered along the microscope’s optical axis and in focus on the objective. Temperature control of the apparatus proved essential to eliminating drift in the optical signal.

**Figure 3.**
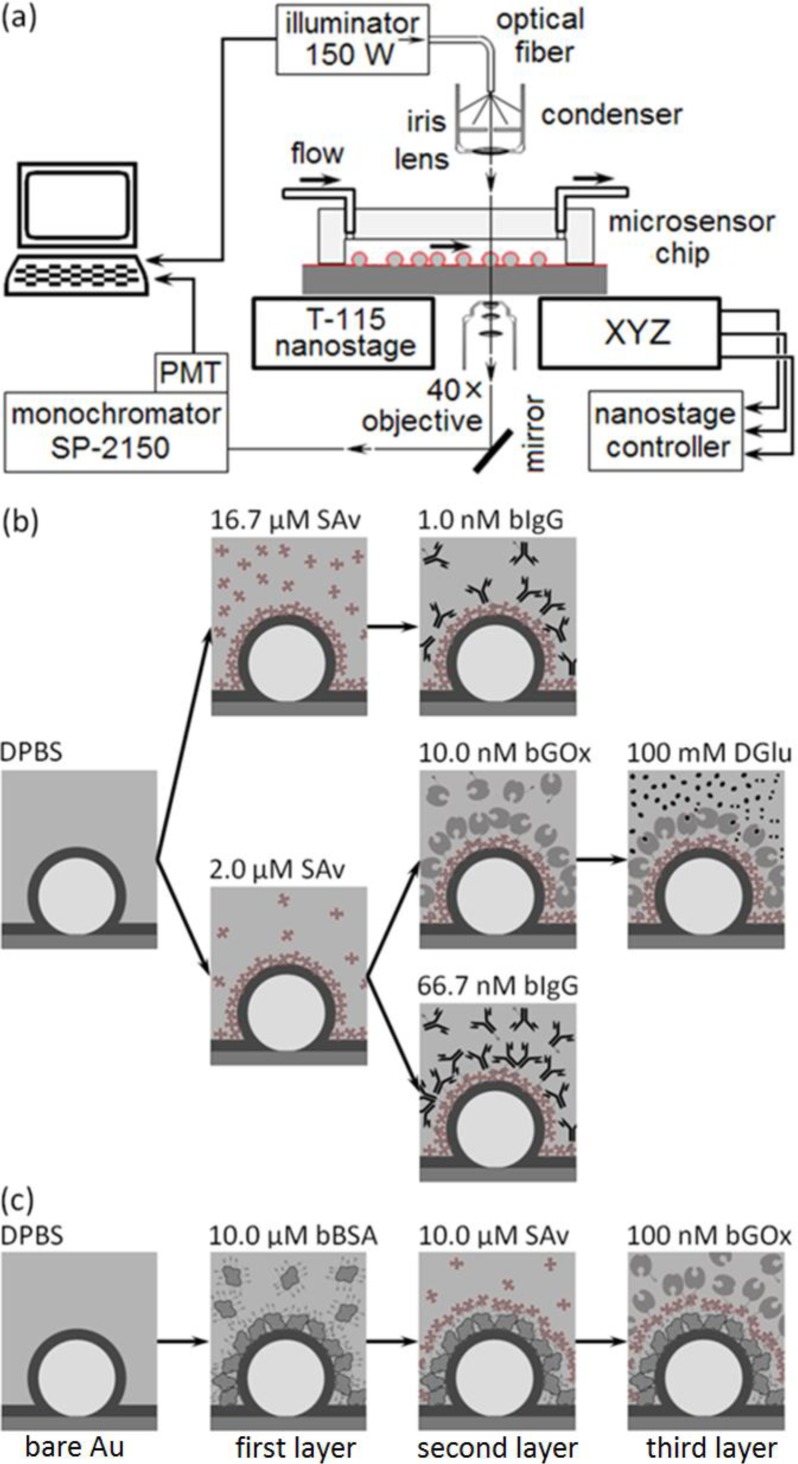
(**a**) Experimental setup: We selected a single microsensor to image using the nano-precision controller stage and analyzed its emitted light using a software-controlled spectrometer. (**b**) Flowchart of a typical target-ligand interaction: the first step shows the formation of the gold-SAv layer, the second the formation of gold-SAv-bIgG and gold-SAv-bGOx layers and the third the interaction of bGOx with DGlu (gold-SAv-bGOx-DGlu). This particular experiment used three different microsensors. (**c**) Flowchart of crosslinker-target-ligand interaction: the first step shows the formation of the gold-bBSA layer, the second the formation of the gold-bBSA-SAv layer and the third the formation of the gold-bBSA-SAv-bGOx layer. In this experiment, the same microsensor monitored all three interactions. Not to scale.

The experimental setup (Figure 3) allowed us to collect both spectra of the microsensor’s visible wavelength emissions (to analyze changes in the microsensor emitted-light intensity due to refractive-index changes in the fluid or to molecular binding to the microsensor surface), and time series of the microsensor’s emission intensity at a fixed frequency (to monitor analyte binding to target proteins anchored to the microsensor surface).

**Figure 4.**
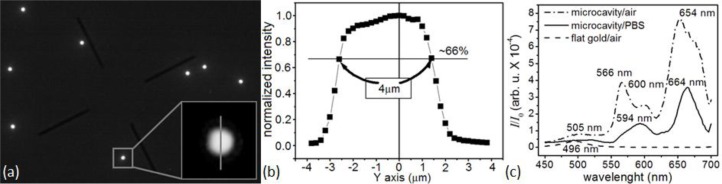
Microsensor characterization: (**a**) White-light transmission micrograph (40×, 0.85 NA) showing eight microsensors randomly distributed over a background of flat gold. The background is dark, showing the enormous level of microsensor light emission compared to the light transmission through the flat gold. (Insert) Microsensor detail: the grey line shows the trajectory of the profile in Figure 4(b). The flat gold film on the glass substrate has a spectral transmission of less than 10^−4^. (**b**) Intensity profile scan across a microsensor (convoluted by the seeing function of the optical apparatus). We aligned the microsensor so the highest intensity point of the microsensor’s emission lay at the origin of the focal axes, moved the nanostage laterally by 4 μm, keeping the focal position fixed, then scanned back along the line indicated in the insert in (a). (**c**) Spectra of the light emitted by a microsensor (a ∅780 polystyrene nanosphere covered with a ≈250 nm gold layer) used in Figure 5(d), in air (dot-dash line) and in PBS (solid line), and the spectrum of the light transmitted through the background flat gold film in air, ≈20 μm away from a microsensor (dash line). In all cases, we excited the microsensor using white light from the microscope illuminator (0.3 NA).

To monitor a microsensor’s emitted light, we custom-modified the stage of an inverted optical microscope (Diaphot, Nikon, Inc.) to accept a T115 Nano-Stage (±10 nm resolution in the *x*, *y* and *z* directions) controlled by a Nano-Drive (Mad City Labs, Inc.). To analyze the light from a single sensor, we used the Nano-Stage to align the microscopes’ optical axis with the microsensor and to bring it into focus, an operation range of ±3 μm in *x* and *y* directions (Figure 4), and ±10 μm in *z* direction. The 40× objective allowed us to select a single microsensor within the acceptance aperture of the spectrometer. Small magnets fixed the microsensor chip to a metal plate on top of the Nano-Stage. We controlled the chip and stage assembly temperature to 24.0 ± 0.1°C.

We illuminated the microsensor chip using a DC-950H Dolan-Jenner DC-Regulated Fiber-Optic Illuminator (Edmund Optics, Inc.). We guided the white light from the illuminator’s 150-W quartz halogen light bulb to the microscope through an optical fiber (0.5 inch core) light pipe, next through an iris, and then refocused it onto the chip using the microscope’s 0.3 *numerical aperture* (NA) condenser. In all experiments we set the lamp intensity to 90% of its maximum intensity. The microscope collected the microsensor’s emitted light through a 40× objective (0.85 NA) and fed it into a spectrometer (300 μm slit-width, 500 nm blaze-wavelength, 600 grooves/mm grating, SpectraPro SP-2150i, Acton Research) through an optical fiber. We mounted one end of the fiber on a 5-degree-of-freedom support in front of a microscope exit port and aligned it with the port’s optical axis. The image of a microsensor at the microscope exit port was less than ∅100 μm (≈0.02 NA) which fit cleanly inside the acceptance window of ∅600-μm of the core optical fiber (0.34 NA). We connected the other end of the optical fiber to the entrance port of the spectrometer. We mounted an integrated photon-counting photo-multiplier tube (*IPC-PMT*, PD-473, Acton Research) at the spectrometer exit slit and used Spectra Sense software (Acton Research) to control the spectrometer’s dispersive element and detector integration time to record both spectra and time-series at fixed wavelengths. A thermo-isolation box housed the microscope, microsensor chip, reagents and buffers. We regulated the temperature inside the box to 24 ± 0.1 °C and the room temperature to 22 ± 1.5°C.

### 2.7. Microsensor Biochemistry

To monitor binding or enzymatic reactions, we had first to attach the target protein under study to the gold surface of the microsensor. We employed two different protocols for microsensor functionalization to determine the degree to which the functionalization details affected detection quality.

**Figure 5.**
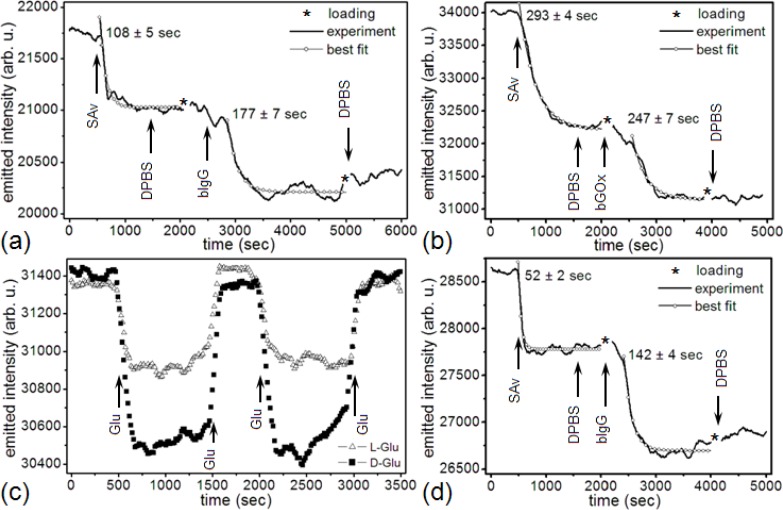
Monitoring the interaction of a target with a specific ligand: (**a**) a microsensor’s emitted-light intensity at 660 nm during the covalent binding of a 16.7 μM SAv solution with a microsensor’s bare gold surface, followed by the interaction of a 1 nM bIgG solution with the microsensor-bound SAv. (◯) exponential-decay best-fits to the emitted-light intensities: *R*^2^(SAv) = 0.88, *R*^2^(bIgG) = 0.84. (**b**) a microsensor’s emitted-light intensity at 660 nm during the covalent binding of a 2 μM SAv solution with the microsensor’s bare gold surface, followed by the interaction of a 10 nM bGOx solution with the microsensor-bound SAv. (◯) exponential-decay best-fits to the emitted-light intensities: *R*^2^(SAv) = 0.99, *R*^2^(bGOx) = 0.96. (**c**) a microsensor’s emitted-light intensity at 660 nm during the interactions of 100 mM DGlu/DPBS (50 mM βDGlu equivalent) and 100 mM LGlu/DPBS solutions with microsensor-bound bGOx. The specific microsensor was that shown in Figure 5(b). The LGlu solution had the same refractive index as the DGlu solution, but did not bind to bGOx. Both DGlu/DPBS and LGlu/DPBS solutions produced fast emitted-light intensity changes on wash-in/wash out because they had a higher refractive index than DPBS. However, only the DGlu/DPBS solution produced a slower change in emitted-light intensity due to formation of βDGlu – bGOx complex. (**d**) a microsensor’s emitted-light intensity at 660 nm during the covalent binding of a 2 μM SAv solution with the microsensor’s bare-gold surface, followed by the interaction of a 66.7 nM bIgG solution with the microsensor-bound SAv. (◯) exponential-decay best-fits to the emitted-light intensities: *R*^2^(SAv) = 0.90, *R*^2^(bIgG) = 0.94. The sample flow rate in all experiments was ≈0.2 nL/s with a solution temperature of 24.0 ± 0.1 °C. * No signal was recorded during sample loading.

In our first experimental protocol (Figure 3(b)), we functionalized the microsensor’s gold surface directly with a target protein, streptavidin (*SAv*, 60 kDa, Sigma-Aldrich, Co.), then monitored the binding of two ligand proteins to the target: biotin-conjugated goat anti-mouse immunoglobulin G (*bIgG*, 150 kDa, Thermo Fisher Scientific Inc.) or biotin-conjugated glucose oxidase (*bGOx*, 160 kDa, Rockland Immunochemicals, Inc.) (Figure 5). We bound SAv to the microsensors, supplying it at either 2 μM (Figures 3(b) and 5(b,d)) or 16.7 μM (1 mg/mL) (Figures 3(b) and 5(a)) in Dulbecco’s Phosphate-Buffered Saline (DPBS, Invitrogen Co.). We next reacted to the bound SAv either bIgG supplied at either 1 nM (Figures 3(b) and 5(a)) or 66.7 nM (1μg/mL) in DPBS (Figures 3(b) and 5(d)), or bGOx supplied at 10 nM in DPBS (Figures 3(b) and 5(b)). Finally, we monitored bGOx interacting with either 100 mM DGlu or 100 mM LGlu (Sigma-Aldrich Co.) both in DPBS (Figures 3(b) and 5(c)).

**Figure 6.**
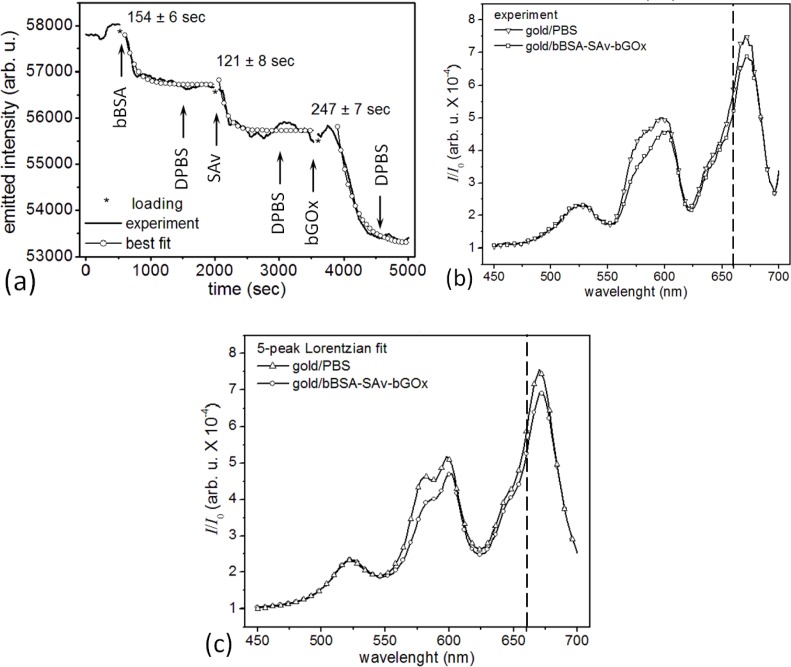
(**a**) Monitoring the successive binding of three molecular layers to a microsensor: a microsensor’s emitted-light intensity at 660 nm (dashed line in Figure 6(b) and 6(c)) during: (i) covalent binding of 10-μM bBSA solution to the microsensor’s bare-gold surface, (ii) subsequent binding of a 10-μM SAv solution to the microsensor-bound bBSA, (iii) subsequent binding of a 100-nM bGOx solution to the microsensor-bound bBSA-SAv complex. (◯) exponential-decay best-fits to the emitted-light intensities: *R*^2^(bBSA) = 0.91, *R*^2^(SAv) = 0.78, *R*^2^(bGOx) = 0.97. The sample flow rate was ≈0.2 nL/s and the solution temperature was 24.0 ± 0.1 °C. *No signal was recorded during sample loading. (**b**) Spectra of a microsensor consisting of a ∅780 polystyrene nanosphere covered uniformly with a ≈150 nm gold film and excited with white light from a microscope illuminator (0.3 NA), showing the four main visible microcavity resonances (i) (▾) spectrum for the bare-gold microsensor in DPBS, (ii) (□) spectrum for the microsensor after deposition of bBSA-SAv-bGOx. (**c**) Multi-peak Lorentzian fits of the spectra in Figure 6(b): (i) (▴) for the bare-gold microsensor in DPBS, *R*^2^(DPBS) = 0.99, and (ii) (◯) for the microsensor after deposition of bBSA-SAv-bGOx *R*^2^(bBSA-SAv-bGOx) = 0.99.

In our second experimental protocol, we functionalized the microsensor’s gold surface with biotin-conjugated bovine serum albumen (bBSA, 66.5kDa, Sigma-Aldrich, Co.) supplied at 10 μM in DPBS, then reacted the bound bBSA with SAv at 10 μM, and finally reacted the bound SAv with bGOx at 100 nM in DPBS (Figures 3(c) and 6).

The first experiment began by loading the end of the reagent tube with the target protein solution. We immediately inserted the tube into the microsensor chip and rinsed with DPBS for 500 s, monitoring the microsensor emissions to define the emission-intensity baseline. We injected the SAv solution and monitored its binding to the gold surface of the microsensor for 1,000 s, then rinsed for 500 s with DPBS. A constant emitted-light intensity during the rinse indicated that a stable molecular layer had formed on the microsensor’s surface. We stopped the recording, unplugged the reagent tube, and rinsed it with DPBS for 10 s, then with 0.1 mM HCl for 10 s and again with DPBS for another 10 s. We then loaded the end of the reagent tube with ≈4 μL of the ligand solution (either bIgG or bGOx), and quickly reconnected the tube to the microsensor chip. During these steps, we continually flushed the microsensor chamber with DPBS from the other input reservoir. We resumed emitted-light intensity recording, injected the ligand into the microsensor chamber, and monitored its binding to the bound SAv on the microsensor for 2,000 s. Once the microsensor’s emitted-light intensity stabilized, indicating that the reaction had saturated, we rinsed the chip with DPBS for 1,000 s. In both cases, the emitted-light intensity remained constant, again indicating that the SAv bound the bIgG and bGOx with very high affinity.

For the bGOx functionality test (Figures 3(b) and 5(b)) in the first experiment, we disconnected the reagent tube and rinsed it as described above, while flushing the microsensor chamber with DPBS from the other input. We then loaded the reagent tube with 20–50 μL DGlu or LGlu solution. We resumed recording the emitted-light intensity, recorded a new baseline for 500 s, then injected the DGlu or LGlu into the microsensor chamber and monitored its enzymatic reaction with the bound bGOx for 1,000 s. To test the repeatability of our enzymatic measurements, we next flushed the microsensor chamber with DPBS for 500 s, then exposed the sensors to DGlu or LGlu solution again and repeated our measurements.

The second experiment was similar, but used three proteins (crosslinker, target and ligand) and DPBS rinsing steps; the protocol employed bBSA rather than SAv in the first step (Figures 3(c) and 6). The last step of the experiment loaded the reagent tube with ≈4 μL of ligand supplied as 100 nM bGOx solution in DPBS, monitoring its interaction with the bound SAv on the microsensor surface.

### 2.8. Data Analysis

We recorded visible-range spectra (450–700 nm) to characterize the microsensor response to changes in the refractive index of the medium and in their surface functionalization. We also recorded time series to characterize specific reactions’ kinetics. We collected spectra at 2 nm resolution with a 1 s detector integration time using 5-point running-average smoothing. We recorded continuous amplitude time series at a fixed 660 nm wavelength using a 5-s detector integration time, with a 70% detector duty-cycle, using 20-point running-average smoothing.

## 3. Results and Discussion

### 3.1. Microsensor Characterization

A microcavity biosensor consists of a gold shell ∅1.05 ± 0.08 μm (*n* = 10) over a dielectric nanocore ( $n_{\mathrm{PS}}^{20}=1.585$ at *λ* = 660nm), with a gap in the gold where the core attaches to a glass surface [110]. When excited with non-polarized white light perpendicular to the glass substrate (Figure 3) the microsensor emits light with a characteristic spectrum dominated by peaks, or *microcavity resonances*, with widths sometimes as narrow as 15 nm [105]. When excited with monochromatic light, the microcavity emission wavelength is the same as the excitation wavelength, with an amplitude depending on the microcavity resonance strength at that wavelength. Refractive index changes in the solution above the microsensor and biomolecular interactions at the microsensor surface change the microcavity resonance widths, strengths and center frequencies [105].

The very weak optical transmission through the bare-flat-gold film covering the glass between the microsensors had a nearly flat spectrum, with a broad transmission peak around 500 nm. The flat gold film thus acted like a neutral density 3 (ND3), with a transmission coefficient of $T_{660\mathrm{nm}}^{\mathrm{air}}=1.4\times 10^{-6}$ of the incident light intensity. The intensity of the light emitted by the microcavity was $\frac{I_{660\;\mathrm{nm}}^{\mathrm{air}}}{I_{0}}=7.1\times 10^{-4}$ of the excitation light intensity, so the background contributed less than 0.2% of the detected light in the recorded signal. The lack of a strong background signal enhanced detection contrast (Figure 4(a–c)) and facilitated microsensor optical alignment and spectral analysis (Figure 4(b,c)). The experiments described in this paper used an optical apparatus that takes advantage of multi-functionality of an inverted microscope; however, preliminary setups (data not shown) have demonstrated that the microsensors can combine with much simpler optics consisting of an inexpensive light emitting diode (LED) light source and a compact charge-coupled device (CCD) or complementary metal-oxide-semiconductor (CMOS) optical detector. Such LED/CMOS device would allow the design of very simple, inexpensive and robust medical instruments based on microsensor chips.

Spectral analysis (450–700 nm) of the light emitted by the microsensors in air (Figure 4(c)) showed four resonances: at 505 nm, 568 nm, 600 nm and 652 nm. When the refractive index increased as biomolecules adsorbed to the microsensor’s surface, the microcavity resonances shifted towards larger wavelengths and decreased in intensity, allowing label-free real-time molecular interaction monitoring [105,106]. The same analysis showed that the 652-nm resonance was the most sensitive to the refractive index change (≈8 nm red-shift, ≈56% amplitude decrease) when we changed the medium in contact with the microsensor switched from air (*n*^20^ = 1.000) to DPBS (*n*^20^ = 1.334). The 652-nm resonance was also the narrowest, with the least overlap with neighboring resonances. It also lay in the red–NIR optical window of tissue transparency, potentially allowing its use for transdermal readout when monitoring biomarkers in body fluids. We therefore used this resonance in our subsequent experiments to monitor reaction kinetics.

Our experiments focused on the microsensor’s responses to ligand concentrations in the 1 nM to 100 nM range which is relevant to both body-fluid biomarker levels and single-cell secretion assays. Like all label-free biosensors, the microsensor’s output signal responds to all refractive index changes at its surface. Since the bare-gold surface of the microsensor would adsorb a wide variety of biomolecules from a complex biofluid, to ensure that the microsensor detects a specific molecule, we must *functionalize* it with a *target* molecule that will bind with very high affinity to the desired *ligand* (to ensure high sensitivity) and with very low affinity to any other biomolecules present in the sample (to ensure high selectivity).

The evanescent SP normal to a metal surface decays exponentially with the normal distance into the dielectric analyte beyond the biosensor's surface; thus changes in the analyte’s index of refraction within one penetration depth of the surface are the primary modulators of the SP resonance. If the penetration depth is of the order of the molecular size of typical analytes, then the SP sensitivity per unit molecular weight will decrease for very large analyte molecules. Similarly, for a multilayer deposited on the biosensor, the sensitivity will decrease for layers lying at distances much beyond a penetration length. However, while a long penetration depth is helpful for measuring the bulk index of refraction of the analyte fluid, it creates an unpredictable signal that reduces the sensitivity of experiments studying specific binding to the surface. A penetration depth comparable to the thickness of the bound complex optimizes such experiments. The penetration depth depends on the excitation wavelength and the biosensor’s material, texture and geometry. For thin, flat gold films, the typical penetration depth is between 150 nm and 450 nm [111], much longer than the typical 10–30 nm size of the analyte complex and too long for optimal signal detection of binding events. Dextran or polymer coated metal surfaces reduce the volume of fluid inside the penetration volume which is not participating in the reaction, thus improving the signal-to-noise ratio. For the nanoparticles the localized SP penetration depth is between 5 nm and 30 nm, comparable to the diameter of the biomolecular complexes under study [112]. This decreased dead volume eliminates the need for a dextran or polymer coating of the microsensor. However, the shorter penetration depth might reduce the sensitivity for very large molecular complexes or for studies of the binding of successive molecular layers, where the final thickness to be detected could be around 30 nm. To check whether the microcavity biosensor penetration depth reduced sensitivity in such experiments, we assessed the microsensor’s response when we sequentially attached two or three layers of large biomolecules to the microsensor, creating biomolecular layers from 10.5 nm to 13.2 nm for the SAv-bGOx layers, from 8.5 nm to 20 nm for the SAv-bIgG layers and from 14.5 nm to 27.2 nm for the bBSA-SAv-bGOx layers.

Our optical apparatus monitored biomolecular interactions at the 660 nm excitation wavelength. Binding of biomolecules at or near the microsensor surface increased the local refractive index, which red-shifted the microcavity resonance frequency and decreased the resonance amplitude (Figure 4(c)). Following the simple kinetic model in [48], we can describe the target-ligand binding kinetics by [106]:

(1) $$R_{\text{ms}}(t)=R_{\text{B}}-\left\{R_{0}+\left[\frac{k_{\text{on}}C}{k_{\text{on}}C+k_{\text{off}}}-R_{0}\right]\times \left\{1-\text{exp}\left[-(k_{\text{on}}C+k_{\text{off}})t\right]\right\}\right\}$$

where *R*_ms_(*t*) is the amount of analyte bound to the microsensor surface at time *t*, *R*_B_ is the instrument baseline, e.g., the refractive index of the rinsing buffer, *R*_0_ is the initial amount of ligand bound at the surface, *C* is the concentration of ligand in solution, and *k*_on_ and *k*_off_ are the *association* and *dissociation kinetic rate constants* respectively. Equation (1) shows that three parameters (*C*, *k*_on_ and *k*_off_) affect molecular binding at the microsensor surface, with a higher value of *k*_on_ increasing the amplitude and speed of response. Therefore, our initial experiments used the biotin-streptavidin receptor-ligand pair because they have the strongest non-covalent binding readily available (dissociation constant, *K*_D_ = *k*_off_/*k*_on_ = 5.3 × 10^−14^ mol/L and *k*_on_ = 7.5 × 10^−7^ L/mol/s [113]). We could then control the interaction rate and amplitude by varying a single parameter, the ligand concentration *C*.

### 3.2. Assessing the Microsensor Response to Two Biomolecular Layers: the Target-Ligand Interaction

The integrity and functionality of both surface-bound target and ligand were crucial since the gold surface induces conformational changes [113] topologically damaging the target’s *paratope* or ligand’s *epitope* compromising the stability of the interaction, reducing their affinity and/or reactivity, thus preventing activity and/or concentration quantification. SAv is a homo-tetramer, 4.5 × 5.0 × 5.5 nm^3^, with four binding sites and multiple disulfide bonds at its surface which bind covalently to gold without requiring additional crosslinkers [114]. When biotin binds to SAv, its active region reaches ≈1.4 nm deep inside the paratope, making its interaction extremely resistant to elution, pH and temperature variations [115]. We tested the functionality of bound SAv by monitoring its interaction with 1 nM bIgG, 10 nM bGOx and 66.7 nM bIgG in DPBS. A constant emitted-light intensity after we rinsed the reacted SAv-bIgG and SAv-bGOx with DPBS indicated that the SAv remained functional and was maintaining a stable interaction with its ligands.

Testing ligand functionality was more complex. Our ligands, bIgG and bGOx have similar molecular masses, but very different shapes. bGOx is a slightly elliptical dimeric protein, 6.0 × 5.2 × 7.7 nm^3^[116] whereas IgG has a tetrameric quaternary structure, 14.5 × 8.5 × 4.0 nm^3^, with two identical heavy chains and two identical light chains, which assembled into a “Y” shape [117]. We chose bGOx to test ligand functionality because it binds small fast-diffusing glucose molecules (180 Da), thus a higher probability to reach the paratope and an advantageous geometrical shape which is hardly hindered by the surface. On the other hand, bIgG binds macromolecules, much larger than glucose; thus the surface could easily hinder their diffusion to bIgG’s paratope [118]. To connect IgG to the gold surface in such a way that each IgG tetramer maintained both paratopes functional, we needed to attach it to the gold surface indirectly via a long crosslinker like protein A or protein G (data not shown). We then tested ligand functionality by monitoring the interaction of SAv-bGOx with 100 mM DGlu (50 mM βDGlu) and 100 mM LGlu in DPBS. The reproducible binding curves indicated that the bound bGOx was enzymatically functional.

Figure 5(a) shows the change in a microsensor’s emitted-light intensity at 660 nm, during the covalent functionalization of the gold surface with ≈16.7 μM (1 mg/mL) SAv [114]. The saturated gold-SAv reaction had a 108 s time constant and decreased the microsensor resonance intensity by 721 counts/s (from a 21,747 counts/s gold/DPBS baseline) with a signal-to-noise ratio (*S/N*) = 17.6 (12.4 dB). The interaction of 1 nM bIgG with surface-bound SAv had a 177 s time constant and decreased the microsensor resonance intensity by 816 counts/s (from a 21,026 counts/s gold-SAv baseline) with a S/N = 14.8 (11.7 dB).

Figure 5(b) shows a microsensor’s emitted-light intensity at 660 nm during functionalization of the microsensor gold surface with 2 μM SAv. The saturated gold-SAv covalent reaction had a 293 s time constant and decreased the microsensor resonance intensity by 1,791 counts/s (from a 34,005 counts/s gold/DPBS baseline) with a S/N = 37 (15.7 dB). The interaction of 10 nM bGOx with surface-bound SAv had a 247 s time constant and decreased the microsensor resonance intensity by 1,071 counts/s (from a 32,214 counts/s gold-SAv baseline) with a S/N = 21.9 (13.4 dB).

Figure 5(d) shows a microsensor’s emitted-light intensity at 660 nm during the functionalization of the gold surface with 2 μM SAv. The saturated gold-SAv reaction had a 52 s time constant and decreased the microsensor resonance intensity by 827 counts/s (from a 28,608 counts/s gold/DPBS baseline) with a S/N = 20.2 (13.0 dB). The interaction of 66.7 nM bIgG with surface-bound SAv had a 142 s time constant and decreased the microsensor resonance intensity by 1,087 counts/s (from a 27,781 counts/s gold-SAv baseline) with a S/N = 21.3 (13.3 dB).

SAv provided a very fast method for preparing a surface for antibody functionalization and its spherical shape produces a compact surface coverage of ≈2.24 fg/μm^2^[114] corresponding to 56,000 SAv molecules on a saturated microsensor. bGOx has an ellipsoidal shape, packing on a smooth gold surface at densities of 2.1–6.1 fg/μm^2^[118–121], corresponding to 19,000 to 57,000 molecules on a saturated microsensor. bIgG has a highly asymmetric shape, packing on a smooth gold surface at a density of ≈1.28 fg/μm^2^[122], corresponding to 13,000 molecules on a saturated microsensor. Numerous factors affect the packing densities achieved by the components during sequential binding of different molecules to form layers on a microsensor: the biosensor surface texture (sputtered-gold is very rough, unlike the evaporated-and-annealed-gold film on a typical SPR chip, which may enhance binding) [105], the method used to attach the first layer of biomolecules to the gold surface (e.g., crosslinkers, buffers, passivators) [118–121,123], and the binding affinities of the components.

A single microsensor required fewer than 56,000 molecules of SAv (≈5.6 fg) to reach a saturated emitted-light intensity with a minimum S/N of 17.9 ± 2.3. The time to saturation was less than 5 min. Considering Poisson noise and neglecting diffusion times in a first approximation, simple *N*^½^ scaling implies a detection limit of 130 to 230 SAv molecules. In the experiment using 10 nM of bGOx binding to SAv, the emitted-light intensity reached saturation in 4 min with a minimal S/N of 21.9, which would imply a detection threshold of 116 molecules. In the experiment using 66.7 nM of bIgG binding to SAv, the emitted-light intensity reached saturation in 2.4 min with a minimum S/N of 21.3, which would imply a detection threshold of 30 IgG molecules. Such results are consistent with our previous findings that microsensor saturation took about 3.5 min and required fewer than 57,000 molecules of GOx (≈9 fg) at a S/N of 50.1, implying a limit of detection of 22 GOx molecules [106]. While many factors influence a microsensor’s effective sensitivity and detection time, e.g., the sizes of both receptor and ligand molecules, their binding affinity, the level and type of nonspecific binding to components in the medium and biosensor’s surface geometry. For most common bioassay molecules assuming physiological concentrations and typical monoclonal antibody affinities, a detection time of less than 5 min for quantitative measurements and less than 1 min for measuring secretion from a single cell should be practical.

SP wave propagation is sensitive to changes in the refractive index, which correlates with the mass of biomolecules bound to the biosensor surface. Classic SPR measures such refractive index changes in *resonance units* (*RU*) [124–126] which scales with biomolecular mass bound to the biosensor surface as 1 RU = 1 pg/mm^2^[127]. The microsensor’s response should scale in a similar way with the molecular mass near its surface. If in a first approximation we assume that the bound molecules are spherical and that both target and ligand are comparable in size, and interact with 1:1 stoichiometry, then for concentric compact monolayers at the microsensor surface, the ratio of the molecules’ molecular weights should equal the ratio of the emitted-light intensities: $\frac{{\mathrm{MW}}_{1}}{{\mathrm{MW}}_{2}}=\frac{{\Delta S}_{1}}{{\Delta S}_{2}}$, where *MW*_1_ and *MW*_2_ are the molecular masses of the target and ligand, and Δ*S*_1_ and Δ*S*_2_ the emitted-light intensity changes on binding of the first and the second molecular layers. Deviations from this predicted ratio then indicate the relative compactness of the bound molecular layers.

Figure 5(a) shows that the gold-SAv – bIgG complex ( $\frac{{\mathrm{MW}}_{\mathrm{SAv}}}{{\mathrm{MW}}_{\mathrm{bIgG}}}=\frac{60\mathrm{kDa}}{150\mathrm{kDa}}=0.4$) produced an emitted-light intensity ratio $\frac{{\Delta S}_{\mathrm{SAv}}}{{\Delta S}_{\mathrm{bIgG}}}=0.88$ (721 counts/s ÷ 816 counts/s). Two compact monolayers would produce an emitted-light intensity ratio $\frac{{\Delta S}_{\mathrm{SAv}}}{{\Delta S}_{\mathrm{bIgG}}}=\frac{1}{2.5}=0.4$, suggesting that the 1 nM bIgG solution did not produce a compact bIgG layer over the previously attached SAv monolayer on the microsensor surface. Figure 5(b) shows that the gold-SAv – bIgG complex ( $\frac{{\mathrm{MW}}_{\mathrm{SAv}}}{{\mathrm{MW}}_{\mathrm{bGOx}}}=\frac{60\mathrm{kDa}}{160\mathrm{kDa}}=0.375$) produced an emitted-light intensity ratio $\frac{{\Delta S}_{\mathrm{SAv}}}{{\Delta S}_{\mathrm{bGOx}}}=1.67$ (1791 counts/s ÷ 1071 counts/s), again suggesting that the 10 nM bGOx solution did not produce a compact bGOx layer over the previously attached SAv monolayer on the microsensor surface. Figure 5(d) shows that the gold-SAv – bGOx complex produced an emitted-light intensity ratio $\frac{{\Delta S}_{\mathrm{SAv}}}{{\Delta S}_{\mathrm{bIgG}}}=0.76$ (827 counts/s ÷ 1087 counts/s), suggesting that again, 66.7 nM bGOx did not produce a compact monolayer on SAv. However, even these sub-monolayer coverages produced a large enough S/N to demonstrate that the microsensor could quantify much smaller concentrations than those used in these experiments.

Figure 5(a,d) clearly shows that after the end of the last rinse step, the microsensor’s emitted-light intensity gradually increased. This gradual increase could indicate either ligand unbinding from the target or conformational changes of the bound ligand. However, Figures 5(b) and 6, which show the same binding protocol using bGOx instead of bIgG, the microsensor’s emitted-light intensity at 660 nm remained constant, showing that the binding of the bGOx was irreversible. Since binding mechanism is the same for bGOx and bIgG, the irreversibility of bGOx binding suggests that bIgG should be similarly irreversible, suggesting that the emitted-light intensity increase results from a conformational change of the bound bIgG. In support of this hypothesis, microcantilever studies have shown that IgG, BSA and Calmodulin gradually change conformation after prolonged contact with a gold surface [128,129].

Figure 5(c) shows the change in a microsensor’s emitted-light intensity when DGlu and LGlu interact with the surface-bound bGOx. The introduction of the DGlu solution into the sample chamber produced a fast decrease in emitted-light intensity because the DGlu solution has a larger refractive index than DPBS. The subsequent, slower change in emitted-light intensity resulted from the formation of βDGlu – bGOx complex. When we rinsed the chip with DPBS, the microsensor’s emission intensity recovered to its former baseline, indicating that the complex was labile. We repeated the DGlu/DPBS, DPBS wash-in/wash-out cycle several times to determine the reproducibility of the detection of complex formation. The cycles produced identical emission dynamics within experimental error. We then ran the same protocol with LGlu to separate the passive effect of refractive index changes in the bulk substrate solutions. Since LGlu solution has the same index of refraction as DGlu solution, but does not complex with bGOx, the difference between the DGlu and LGlu wash-in/wash-out emitted-light intensities represented the complexing of DGlu with bGOx. The LGlu solution produced the same rapid changes in emitted- light intensity we had observed for DGlu on wash-in and wash-out, followed by stable emitted-light intensities for both DPBS and LGlu solution, indicating the absence of both specific and non-specific interactions between the LGlu and the sensor. This results are consistent with our previous findings [106], suggesting that the microsensor-bound bGOx molecules remained enzymatically functional and retained their ability to selectively bind DGlu rather than LGlu.

### 3.3. Assessing the Microsensor Response to Triple Biomolecular Layers: The Crosslinker-Target-Ligand Interaction

Measuring the correlated activity of a pair of target biomolecules that bind specifically to different epitopes of a specific ligand increases both the sensitivity and selectivity of detection of the ligand compared to detection using a single target. Usually, the first target is fixed to the biosensor surface and captures, with high affinity, both the ligand and some distribution of other biomolecules in the sample medium which bind either nonspecifically to the biosensor or to the first target with lower affinity. These bound non-ligand molecules generate a measurement error. After the initial binding has run to saturation, introducing a second target in solution which binds with very high affinity to a different epitope of the sensor-bound ligand will produce a signal which depends almost entirely on the amount of sensor-bound ligand. The enhanced specificity arises because the second target molecule is highly unlikely to have significant accidental affinities for non-ligand molecular species as the first target and thus is unlikely to bind to any sensor-bound molecules except the ligand. The second target can also have a tag or a label amplifying the signal from the secondary interaction (as in ELISA), thus improving the sensitivity of ligand detection. One complication of such dual-target assays is the more complex wash-in/wash-out sequence that increase the measurement time and prevent truly continuous concentration monitoring, though repeating the measurement cycle can provide a series of snapshots of instantaneous concentrations. If the measurement time is short compared to the timescale over which the ligand concentration changes, such repeated measurements may suffice to monitor the ligand concentration kinetics. In addition, because the microsensor responds primarily to molecules within the plasmon electric field penetration depth from the microsensor surface, we were concerned that the microsensor might be less sensitive to the binding of a third molecular layer. To test the feasibility of multiple biomolecular layers with our microsensors, we assessed the sensitivity and selectivity of a microsensor to the successive binding of three biomolecular species. Figure 6(a) shows the change in a microsensor’s emitted-light intensity at 660 nm when we *sandwiched* SAv between bBSA and bGOx. We first functionalized the bare-gold surface of a microsensor with a crosslinker biomolecule, bBSA at 10 μM. We then monitored the specific binding of the target, SAv at 10 μM, binding to the sensor-bound bBSA. Finally, we monitored the specific binding of the ligand, GOx at 100 nM, to the bBSA-bound SAv. bBSA provides an alternative to SAv to prepare a microsensor surface for further functionalization. BSA is an ellipsoidal protein (4 nm × 14 nm) with 16 disulfide bonds that can covalently bind a gold surface with a coverage density of ≈1.21 fg/μm^2^ (or 28,000 BSA molecules for saturated coverage of a microsensor). Each conjugated bBSA molecule has eight to 16 biotin tethers, which could bind 2–3 SAv molecules [122,130,131]. bBSA and SAv have different shapes, but similar molecular weights, about 2.7 time lighter than bGOx.

The covalent binding of bBSA to the bare-gold microsensor had a 154 s time constant and decreased the microsensor’s emitted-light intensity at 660 nm by 1,140 counts/s (from 57,870 counts/s for bare gold in DPBS) with a S/N = 15.6 (12 dB). The binding of SAv to the microsensor-bound bBSA had a 121 s time constant and decreased the microsensor’s emitted-light intensity at 660 nm by 1,000 counts/s (from 56,730 counts/s for the bBSA-covered microsensor) with a S/N = 9.2 (9.6 dB). The binding of bGOx to the bBSA-bound SAv had a 247 s time constant and decreased the microsensor’s emitted-light intensity at 660 nm by 2,459 counts/s (from 55,730 counts/s for the SAv-bBSA-covered microsensor) with a S/N = 23.6 (13.7 dB).

Protein binding near the microsensor’s surface increased the refractive index within the plasmon’s penetration depth, increasing the optical path of the microcavity resonances, red shifting them and decreasing their amplitude. Figure 6(b) compares the microsensor’s emitted-light spectra for bare gold in DPBS, and after coverage with bBSA-SAv-bGOx. A multi-peak Lorentzian analysis (Figure 6(c)) of the spectra in Figure 6(b) showed that the bare gold microsensor in DPBS had four microcavity resonances in the visible range: *λ*_1_ = *λ̄*_1_ ± δ*λ*_1_ = 578.6 ± 0.8 nm, *I*_1_ = 3.533; *λ*_2_ = 599.8 ± 0.6 nm, *I*_2_ = 3.958; *λ*_3_ = 643.0 ± 1.0 nm, *I*_3_ = 1.984; and *λ*_4_ = 670.8 ± 0.3 nm, *I*_4_ = 7.306. After saturation with bBSA-SAv-bGOx (Figure 6(a)) the microsensor’s four microcavity resonances shifted to: *λ*′_1_ = 581.8 ± 1.3 nm, *I*′_1_ = 3.075; *λ*′_2_ = 601.6 ± 0.6 nm, *I*′_2_ = 3.504; *λ*′_3_ = 644.1 ±1.0 nm, *I*′_3_ = 2.053; and *λ*′_4_ = 672.0 ± 0.3 nm, *λ*′_4_ = 6.653. As before [106], we used the forth resonance to monitor interaction kinetics because it had the largest relative drop in amplitude on binding *λI*_4_ = 0.653 and the least overlap with the other resonances. The light transmitted through the flat gold film surrounding the microsensor had a weak spectral maximum at *λ*_0_ = 521.3 ±1.1 nm, *I*_0_ = 2.044 for bare gold in DPBS, and at *λ*′_0_ = 522.3 ± 1.1 nm, *λ*′_0_ = 2.093 after the binding of the bBSA-SAv-bGOx multilayer; the bBSA-SAv-bGOx multilayer binding had negligible effect on the flat-gold transmission spectrum or amplitude (Δ*λ*_0_ = 1.0 ± 2.2 nm, Δ*I*_0_ = 0.049).

Under the same hypothesis that the ratio in change in a microsensor’s emitted-light intensity equals the ratio of the molecular masses of the corresponding layers bound to the microsensor, $\frac{{\mathrm{MW}}_{1}}{{\mathrm{MW}}_{2}}=\frac{{\Delta S}_{1}}{{\Delta S}_{2}}$, Figure 6(a) shows that gold-bBSA – SAv complex ( $\frac{{\mathrm{MW}}_{\mathrm{bBSA}}}{{\mathrm{MW}}_{\mathrm{SAv}}}=\frac{66\mathrm{kDa}}{60\mathrm{kDa}}=1.1$) produced an emitted-light intensity ratio $\frac{{\Delta S}_{\mathrm{bBSA}}}{{\Delta S}_{\mathrm{SAv}}}=1.14$ (1,140 counts/s ÷ 1,000 counts/s), suggesting that the 10 μM SAv solution formed a compact monolayer of SAv on top of the microsensor-bound bBSA. The next layer, gold-bBSA-SAv – bGOx complex ( $\frac{{\mathrm{MW}}_{\mathrm{SAv}}}{{\mathrm{MW}}_{\mathrm{bGOx}}}=\frac{60\mathrm{kDa}}{160\mathrm{kDa}}=0.38$) produced an emitted-light intensity ratio $\frac{{\Delta S}_{\mathrm{SAv}}}{{\Delta S}_{\mathrm{bGOx}}}=0.40$ (1,000 counts/s ÷ 2,459 counts/s), suggesting that 100 nM bGOx solution again produced a compact monolayer of bGOx on the layer of microsensor-bound bBSA-SAv. Again, the recorded S/N would allow quantification of much smaller concentrations of reactants than used in these experiments.

## 4. Conclusions/Outlook

Personalized medicine’s key need is to be able measure conveniently, flexibly and affordably, the kinetic responses of large panels of biomarkers simultaneously in body fluids in real-time. Since biomarker levels span an incredible range of concentrations from 0.1 pM to 10 mM, a biosensor with great sensitivity over a limited range of concentrations is less useful clinically than one with adequate sensitivity over a broad dynamic range. Additional factors that may be more important than absolute sensitivity, including biosensor size, fabrication complexity, operating life, shelf life, and the complexity and operating cost of the instrument which interrogates the biosensor. The microcavity biosensors we have presented meet all of these requirements. They are small, ∅1 μm, simple and inexpensive biosensors that can dynamically detect, quantify and monitor biomolecular interactions over a broad range of physiologically relevant concentrations. While we have optimized the microsensors primarily for the single-target, label-free detection which is most convenient for high-throughput dynamic measurement of large panels of biomarkers, we have also shown that the microsensors would work effectively with multiple biomolecular layers to maximize selectivity. Because the amount of reagent the microsensors require for molecular quantification is very small (<<20,000–60,000 molecules) they could also be integrated into micro-wells for detection of molecular species secreted by individual cells.
